# Hair cell loss – cause or consequence of hearing loss?

**DOI:** 10.3389/fcell.2026.1777308

**Published:** 2026-04-09

**Authors:** Maurizio Cortada, Soledad Levano, Daniel Bodmer

**Affiliations:** 1 Department of Biomedicine, University of Basel, Basel, Switzerland; 2 Clinic for Otorhinolaryngology, Head and Neck Surgery, University of Basel Hospital, Basel, Switzerland

**Keywords:** cause, cochlea, hair cell, hair cell loss, hearing loss, stereocilia degeneration, strial atrophy, synaptopathy

## Abstract

Hair cell loss is a major hallmark of hearing loss. Historically, hair cell loss has been considered the major cause of hearing loss. Typically, synapses between hair cells and auditory nerve fibers and stereocilia are lost even before hair cells. Loss of these structures is often irreversible leading to permanent hearing loss. The earlier we can intervene in the course of hearing loss, the better we can maintain hearing function. Therefore, it is of importance to precisely understand the underlying causes and sequence of events leading to hearing loss. Notably, hearing loss precedes hair cell loss in many cases. Upon hearing loss, hair cells are present but exhibit morphological and functional defects. Thus, hair cell loss could merely be a secondary consequence of the loss of function of these sensory cells. Here, we review the literature highlighting that hair cell loss is often secondary to hearing loss. We suggest that hair cell loss is often rather the consequence than the cause of hearing loss.

## Introduction

Hearing loss most frequently originates from the inner ear, that is, from sensorineural structures. Sensory hair cells of the inner ear convert mechanical sound waves into electrical signals. These hair cells have synaptic contacts with auditory nerve fibers, which transmit the signal to the central auditory system. Hair cell loss is a major hallmark of sensorineural hearing loss. It can be observed in many hearing loss forms. Based on this observation, historically, loss of hair cells has been considered a major contributor to hearing loss. With advances in the field, synaptopathy has become widely recognized as a cause of hearing loss without hair cell loss. There is now substantial evidence that synapse loss between hair cells and auditory nerve fibers can occur prior to, or in the absence of, overt hair cell loss (Kujawa and Liberman, 2006; Stamataki et al., 2006; Kujawa and Liberman, 2009; Furman et al., 2013; Sergeyenko et al., 2013; Liberman and Kujawa, 2017). This cochlear synaptopathy has been mostly linked to age-related and noise-induced hearing loss. In addition, other mechanisms independent of hair cell loss–including morphological changes in hair cells, metabolic dysfunction, and pathology of surrounding supporting cells (reviewed in (Wan et al., 2013; Wang and Puel, 2018)) – have been recognized as contributors to hearing loss. Despite these advances, it remains unclear to what extent hair cell (and synapse) loss represent primary drivers of auditory dysfunction versus downstream consequences of other functional deficits.

Hearing loss is a major public health concern. It is considered the most frequent sensory deficit. According to the World Health Organization, 5% of the global population suffer from disabling hearing loss that requires rehabilitation (WHO, 2021). Unaddressed hearing loss places a significant burden on those affected, leading to social isolation among others (WHO, 2021). Hearing loss also has a substantial socioeconomic impact on our society (WHO, 2021). Moreover, hearing loss is the largest modifiable risk factor for developing dementia (Livingston et al., 2020). Despite the high prevalence and impact of hearing loss, treatment options are limited to prosthetic devices. Thereby, hearing can only be restored with hearing aids, or cochlear implants in severe hearing loss cases. Recently, the antioxidant sodium thiosulfate has been approved to prevent hearing loss in children receiving cisplatin to combat solid tumors, a chemotherapeutic agent with ototoxic side-effects (Freyer et al., 2017; Brock et al., 2018). Currently, gene therapies are being developed for the treatment of genetic hearing loss and first human clinical trials are ongoing (Lv et al., 2024; Valayannopoulos et al., 2025). Notably, for gene therapy to be efficient, it needs to take place before hair cell degeneration. Unless these aforementioned therapies, no other medical therapies are available for the treatment of hearing loss. To develop novel therapies against hearing loss, it is essential to understand the underlying causes, but also the course of the disease and affected structures.

Hearing loss can be either genetic or acquired. At least 156 non-syndromic hearing loss genes have been discovered to date (Walls et al., 2026), and around 400 syndromic hearing loss genes (Michalski and Petit, 2022). Many of these genes are hair cell genes. The study of these genes in mouse models has informed us much about the molecular composition and functioning of inner ear cells (Michalski and Petit, 2022). Causes of acquired hearing loss include exposure to noise, ototoxic drugs, infections, and aging. Notably, hair cells are often affected and lost in acquired hearing loss.

**FIGURE 1:**
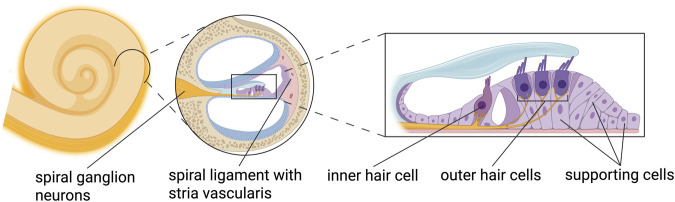
The cochlea and structures essential for hearing function.

In the mammalian cochlea, there are two types of hair cells: inner hair cells and outer hair cells (Figure 1). Inner hair cells are the actual sensory cells, they have afferent synaptic contacts with the auditory nerve formed by spiral ganglion neurons to propagate all auditory information. Outer hair cells are electromotile and amplify the auditory signal to improve sensitivity and frequency selectivity. Different types of supporting cells are also critical for normal hair cell function. The stria vascularis produces the endocochlear potential that is required for mechanoeletrical sound transduction in hair cells and is therefore referred to as the cochlear battery.

While all of these cochlear structures are implicated in hearing, hair cells are among the most vulnerable cells and often lost early in hearing loss. Commonly, hair cells in the high frequency region are lost at first and outer hair cells are more vulnerable than inner hair cells. The reason for this tonotopic and cell type specific vulnerability is not clear. Several mechanisms have been proposed, such as differential distribution of antioxidants (Sha et al., 2001), clock proteins (Park et al., 2017) or calcium homeostasis (Fettiplace and Nam, 2019). Cell type-specific and tonotopic gradients of metabolic processes might also play a role (O'Sullivan et al., 2023).

In our recent study, where we were elucidating the cause of hearing loss in a genetically modified mouse, we found extensive hair cell loss that occurred only after the onset of hearing loss (Cortada et al., 2023). Instead, hearing loss appeared to be due to dysfunctional hair cells resulting from stereocilia pathology and synapse loss, with hair cells degenerating only secondarily (Cortada et al., 2023). While searching the literature, we found that this is common to many hearing loss forms. In this review, we would like to explore the hypothesis that hair cell loss may be a result of the functional decline of these cells, and thus merely a consequence of hearing loss rather than its primary cause. A first section will address this in the context of genetic hearing loss while in a second section we will focus on acquired forms of hearing loss. Finally, we will discuss evidence from human studies and evidence where hair cell loss might be a primary cause of hearing loss.

### Genetic hearing loss and hair cell loss

Genetic models of hearing loss are well suited to determine whether impaired hair-cell function directly leads to hair-cell degeneration. Thereby, mouse models with mutations in genes affecting hair cell function can be assessed for the occurrence (and sequence) of hair cell loss and hearing loss.

The mouse has proven to be a useful tool for studying mechanisms of hearing (loss) by identifying the role of specific proteins whose gene mutations lead to human deafness and have been recapitulated in the mouse (Brown et al., 2008). The first deafness genes discovered occurred as spontaneous mutations in mice and were later found to also be involved in human hereditary hearing loss (Carlson and Avraham, 2022). This review is not intended to give a complete overview of deafness mouse models. Rather, we would like to discuss a number of mutations affecting different hair cell and cochlear functions and their influence on hair cell survival.

In 1980, Karen Steel and Gregory Bock challenged the assumption that hereditary deafness is always caused by the absence of hair cells or their rapid death. They noted that in *deafness* mice, which are profoundly deaf at birth and never develop any auditory function, hair cells are present at young ages and only degenerate progressively with age, suggesting that the loss of hair cells was a secondary consequence of the mutation (Steel and Bock, 1980). The underlying mutation in *deafness* mice was later shown to be in the transmembrane channel-like 1 (*Tmc1*) gene (Kurima et al., 2002), which is a pore-forming subunit of the mechanoelectrical transduction (MET) channel of hair cells (Kawashima et al., 2011). Earlier than hair cell loss in *deafness* mice are some cytological abnormalities (Bock and Steel, 1983; Pujol et al., 1983). Notably, in *Tmc1/Tmc2* knockout mice the hair cell stereocilia bundle starts to degenerate at early postnatal ages, but only after the hair cell mechanotransduction current has been abolished (Kawashima et al., 2011). This is because MET channel function regulates stereocilia length (Velez-Ortega et al., 2017) and shapes stereocilia during development (Krey et al., 2020; McGrath et al., 2021; Krey et al., 2023).

Similarly, *Shaker-1* mice, which are known for vestibular deficits and deafness (Lord and Gates, 1929), have an underlying mutation in the *Myo7A* gene encoding the unconventional Myosin-VIIA protein (Gibson et al., 1995). *Shaker-1* mutant mice gradually lose their hearing (Lord and Gates, 1929; Self et al., 1998; Amariutei et al., 2025), while hair cells are still present (Self et al., 1998; Amariutei et al., 2025). MYO7A regulates stereocilia bundle structure and integrity (Self et al., 1998; Amariutei et al., 2025; Underhill et al., 2025). In humans, mutations in *MYO7A* are responsible for a subtype of Usher syndrome (Weil et al., 1995), which is characterized by hearing loss and vision loss (retinitis pigmentosa), and sometimes balance disorders (Petit, 2001). Usher syndrome type 1 is molecularly characterized by defects in stereocilia (Petit, 2001; Richardson et al., 2011; Michalski and Petit, 2015). Stereocilia are the hair-like projections extending from the Apex of hair cells, arranged in three rows of increasing height and connected to each other by so called tip links (Michalski and Petit, 2015). Stereocilia are the “hearing sensors”, containing the MET channels, and thus form the structure where mechanoelectrical transduction occurs. Usher syndrome type 1 mouse mutants show all stereocilia morphological defects, accounting for the hearing loss (Lefevre et al., 2008; Richardson et al., 2011) and hair cells degenerate only after hearing loss (Lentz et al., 2013). In two further mouse mutants, *Ames waltzer* and *waltzer* mice, the causative mutations were identified in the genes encoding the proteins protocadherin-15 and cadherin-23, respectively (Alagramam et al., 2001; Di Palma et al., 2001). Protocadherin-15 and cadherin-23 are the proteins forming the tip-links, which connect adjacent stereocilia rows; stereocilia deflection tensions the tip links to gate MET channels (Bartsch et al., 2019; Caprara and Peng, 2022). Accordingly, *A. waltzer* and *waltzer* mice, which are characterized by deafness and vestibular disorders, showed stereocilia defects leading to congenital hearing loss, whereas hair cell loss only appeared secondarily (Alagramam et al., 2001; Di Palma et al., 2001). Apart from tip links, other links interconnect stereocilia during development, such as ankle links. When ankle links fail to form, mice show disorganized hair bundles, impaired mechanotransduction and early hearing loss, whereas hair cells are only lost later with age (McGee et al., 2006). To conclude, the study of genetic mouse models not only delineated molecular components and function of the hair bundle, but also demonstrated that structural loss of stereocilia leads secondarily to dysfunctional hair cells and hair cell loss.

In addition to stereocilia, another essential structure for hair cell function and hearing are the inner hair cell synapses. These synapses confer all auditory information via auditory nerve to the brain. To achieve the considerable sustained, rapid and temporally precise hearing performance, inner hair cell synapses have a unique molecular composition (reviewed in (Safieddine et al., 2012; Johnson et al., 2019)). Sound induced depolarization of inner hair cells leads to calcium entry at voltage-activated Cav1.3 (alpha 1D) channels (Platzer et al., 2000), coupling depolarization to neurotransmitter release. The calcium sensor Otoferlin mediates the fusion of presynaptic vesicles with the plasma membrane in inner hair cells (Roux et al., 2006; Michalski et al., 2017), involving soluble N-ethylmaleimide-sensitive factor attachment protein receptor (SNARE) complex proteins such as synaptosome-associated protein of 25 kDa (SNAP-25) (Calvet et al., 2022). The loading of synaptic vesicles with glutamate depends on the Vesicular glutamate transporter 3 (VGLUT3) in inner hair cells (Seal et al., 2008). Pathogenic mutations in the genes encoding these proteins cause deafness due to unfunctional synaptic transmission in inner hair cells. *Cav1.3* (alpha 1D) knockout mice are deaf already at hearing onset, whereas inner hair cells show an initial normal morphology and degenerate over time (Platzer et al., 2000; Glueckert et al., 2003; Dou et al., 2004). Notably, in these *Cav1.3* knockout mice outer hair cells in the cochlear Apex degenerate before inner hair cells (Platzer et al., 2000; Glueckert et al., 2003; Dou et al., 2004). Otoferlin (*Otof*) knockout mice are profoundly deaf, but have a normal development of inner hair cell synapses and show intact hair cells at adult ages (Roux et al., 2006). With increasing age, *Otof* knockout mice show reduced synapse numbers (Stalmann et al., 2021). With further increasing age, inner hair cells are gradually lost (Roux et al., 2006; Stalmann et al., 2021). Interestingly, also outer hair cells showed accelerated degeneration in *Otof* knockout mice when compared to control mice (Stalmann et al., 2021). Similarly, when SNAP-25 protein function is disrupted in inner hair cells after hearing onset, mice exhibit impaired exocytosis and hearing loss, followed by progressive synapse degeneration and ultimately inner hair-cell loss (Calvet et al., 2022). *Vglut3* knockout mice are also profoundly deaf at hearing onset, with no hair cell loss but altered synapse morphology and also reduced synapse numbers with increasing age (Ruel et al., 2008; Seal et al., 2008). *Vglut3* knockout mice retain normal hair cell numbers until at least 20 weeks of age, whereas at 32 weeks of age reduced inner hair cell numbers are observed in mid-basal cochlear regions (Zhao et al., 2025). In contrast, there is no increased outer hair cell degeneration in *Vglut3* knockout mice when compared to control mice with increasing age (Zhao et al., 2025). In summary, impaired synaptic functions lead to the degeneration of synapse numbers over time, followed by hair cell death.

Outer hair cells are an evolutionary innovation of mammals, and their electromotility ensures an exceptional hearing sensitivity and frequency selectivity in mammals (Franchini and Elgoyhen, 2006; Elgoyhen and Franchini, 2011). The protein responsible for outer hair cell electromotility is PRESTIN (Zheng et al., 2000). Prestin (*Slc26a5*) knockout leads to loss of electromotility in outer hair cells and an increase in hearing threshold of around 40–60 dB SPL (Liberman et al., 2002; Dallos et al., 2008). Liberman et al. noted outer hair cell loss in *Slc26a5* knockout mice (Liberman et al., 2002). Notably, Wu et al. precisely investigated the occurrence of hair cell structural abnormalities, hair cell loss and hearing loss in *Slc26a5* knockout mice (Wu et al., 2004). *Slc26a5* knockout mice have already elevated hearing threshold around hearing onset when compared to control mice (Wu et al., 2004). However, around this age, outer hair cells do not show any structural abnormalities (Wu et al., 2004). In addition, there is no significant hair cell loss in hearing impaired mice at young ages (Wu et al., 2004). With increasing age, however, *Slc26a5* knockout mice showed first outer hair cell loss, then inner hair cell loss, pronounced in the basal cochlear turn and involving apoptotic cell death mechanisms (Wu et al., 2004). Similarly, a *Prestin* knockin (KI) mouse model generated to impair PRESTIN function without altering outer hair cell mechanical properties displayed progressive hair cell loss over age, but only after outer hair cells were unfunctional (Cheatham et al., 2015). Therefore, loss of outer hair cell function leads to hearing loss and a secondary degeneration of outer and inner hair cells over time.

What about defects other than in sensory hair cells that are expected to impair hair cell function? There are different types of supporting cells that surround the sensory hair cells in the cochlea (Figure 1). The supporting cells are responsible for important functions in the cochlea such as assuring structural integrity, homeostasis of ions and small molecules, and modulation of the extracellular matrix among others (Wan et al., 2013). It is therefore evident, that although not sensory by nature, defects in supporting cells can cause hearing loss by affecting hair cell function. For instance, to regulate ion homeostasis, supporting cells (but not hair cells) express different types of connexin proteins, which form gap junctions. *Connexin 26* (*Cx26*, GJB2) mutations underly the most common form of genetic hearing loss (Kelsell et al., 1997). Most *Cx26* mutations are thought to impair gap junction function and ion homeostasis in supporting cells (Wingard and Zhao, 2015). Over 450 pathogenic variants have been described for the *Cx26* gene, leading to different hearing loss phenotypes (Wingard and Zhao, 2015; Mao et al., 2023). The precise molecular mechanisms underlying hearing loss in *Cx26* mutations are not precisely understood. Although some early studies suggested cellular (including hair cell) degeneration in *Cx26* knockout mice, Zhao and colleagues showed that hair cell degeneration is not the primary cause of hearing loss in Cx26 deficient mice (Liang et al., 2012; Wingard and Zhao, 2015). Rather, relevant hair cell loss was not visible until adult ages, whereas hearing loss was already present at postnatal ages (around hearing onset) (Liang et al., 2012; Wingard and Zhao, 2015). Nevertheless, the pathogenic mechanism in *Cx26* knockout models is almost certainly influenced by the particular mutation studied, and Cx26 has additionally been implicated in key aspects of cochlear development (Wingard and Zhao, 2015).

The stria vascularis is another essential cochlear structure for hearing. The stria vascularis secrets potassium ions into the endolymphatic space, where the stereocilia are bathing (Hibino and Kurachi, 2006). The remarkably high potassium concentration in the endolymph is maintained by the stria vascularis and also named the endocochlear potential. It allows for depolarization of hair cells after potassium influx at the MET channels upon sound induced stereocilia deflection. A voltage-gated potassium channel in the stria vascularis responsible for secreting potassium ions into the endolymph is formed by subunits KCNQ1 and KCNE1 (Hibino and Kurachi, 2006). Mutations in the genes of either subunit can cause Jervell and Lange-Nielsen syndrome, characterized by sensorineural hearing loss and long QT syndrome (Lee et al., 2000; Casimiro et al., 2001; Hibino and Kurachi, 2006; Chang et al., 2015). Mutations in the *Kcnq1* gene, exclusively expressed in stria vascularis in the inner ear, cause hearing loss in mice (Lee et al., 2000; Casimiro et al., 2001; Chang et al., 2015). An initial study documented hair cell loss in *Kcnq1* knockout mice (Lee et al., 2000). However, a follow-up study looking at temporal occurrence of morphological changes, showed that hair cell loss occurred only after hearing loss in *Kcnq1* knockout mice (Casimiro et al., 2001). Notably, gene therapy not only restored hearing in *Kcnq1* knockout mice, but could also prevent hair cell loss (Chang et al., 2015). Similarly, in a mouse model for Norrie disease, a genetic deaf blindness disorder, vascular abnormalities in the stria vascularis led to a reduced endocochlear potential and preceded hair cell loss (Bryant et al., 2022). The rescue of the vascular pathology in the stria vascularis was able to prevent hair cell loss (Patel et al., 2024), confirming that hair cell loss occurs secondarily in this disease model and restoration of cochlear function can prevent hair cell loss.

Collectively, these studies indicate that defects in genes regulating hair cell function lead to hearing loss that precedes hair cell loss. Functional and morphological alterations in hair cells occur early and likely underlie the initial decline in auditory function, whereas hair cell degeneration appears as a secondary event. Importantly, this sequence is observed not only in mutations affecting hair cell–intrinsic genes, but also in defects in other cochlear components—such as supporting cells or the stria vascularis—that indirectly compromise hair cell function.

### Acquired hearing loss and hair cell loss

In the case of acquired hearing loss, it is more difficult to determine the exact sequence and initial cause of hearing loss, as various pathological mechanisms are likely to be involved, possibly simultaneously. Age-related hearing loss is considered the most frequent sensory deficit, and affects humans and mice alike. For this reason, the mouse once again presents itself as a suitable model for understanding the mechanisms of age-related and other forms of acquired hearing loss. A study by He and colleagues performed a precise molecular and cytological analysis of aging hair cells (Liu et al., 2022). With increasing age, hair cells showed a reduction in cell size as well as stereocilia degeneration (Liu et al., 2022). Outer hair cells displayed reduced axial stiffness with aging, and functionally showed reduced electromotility (Liu et al., 2022). Transcriptional analysis revealed reduced expression of numerous genes with age that are relevant for hair cell function, such as synaptic transmission, mechanotransduction, and electromotility (Liu et al., 2022). Overall, this study revealed that hair cells undergo significant ultrastructural and functional decline prior to hair cell loss, giving pathophysiologic insights into age-related hearing loss (Liu et al., 2022). Notably, prior studies highlighted a reduction of PRESTIN protein amount in ageing outer hair cells, together with a reduction in cell surface area, which was not a sign of apoptosis (Bai et al., 2019; Jeng et al., 2020; Zhang et al., 2022) and occurred markedly prior to outer hair cell loss (Jeng et al., 2020; Zhang et al., 2022). Together, these results reveal functional changes in aging (outer) hair cells, which occur prior to hair cell loss.

**FIGURE 2:**
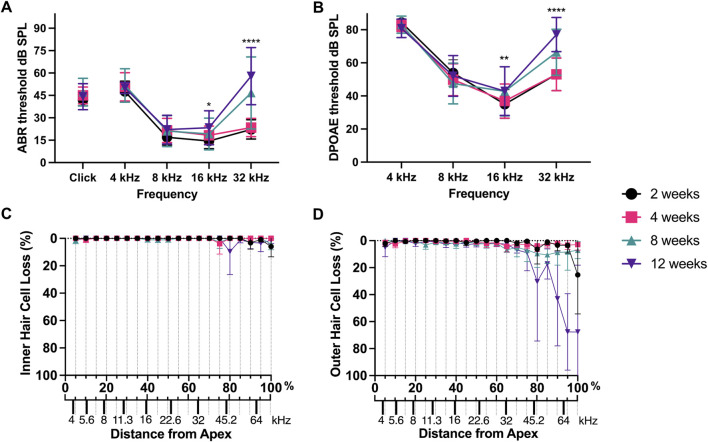
ABR and DPOAE hearing thresholds as well as hair cell quantification in aging C57BL/6J mice. Data re-analyzed from Cortada et al. (2023). **(A)** ABR thresholds measured for a click stimulus or frequencies indicated at 2 weeks (n = 18 ears from 9 animals), 4 weeks (n = 26 ears from 13 animals), 8 weeks (n = 18 ears from 9 animals), and 12 weeks of age (n = 12 ears from 6 animals). Results are presented as means ± SDs. One-way ANOVA followed by a Tukey’s multiple comparison’s test, *p < 0.05 12 weeks vs. 2 weeks at 16 kHz, ****p < 0.0001 8 and 12 weeks vs. 2 weeks at 32 kHz. **(B)** DPOAE thresholds measured for frequencies indicated at 2–12 weeks of age in ears/animals indicated in **(A)**. Results are presented as means ± SDs. One-way ANOVA followed by a Tukey’s multiple comparison’s test, **p < 0.01 12 weeks vs. 2 weeks at 16 kHz, ****p < 0.0001 12 weeks vs. 2 weeks at 32 kHz, **p < 0.01 8 weeks vs. 2 weeks at 32 kHz. **(C,D)** Cochleograms showing inner hair cell (left graph) and outer hair cell (right graph) loss in 5 percent (%) distances from the Apex at indicated ages. Place-frequency map calculated with formula d = (LOG10 ((f+6.664)/9.8)/LOG10(10))/0.0092 (where d is the distance from the Apex in % and f the frequency in kHz). n = 3-4 mice per genotype. Results are presented as means ± SDs.

The widely used C57BL/6J mouse strain carries a single-nucleotide polymorphism in the *Cdh23* gene (Noben-Trauth et al., 2003), making these mice more susceptible to age-related hearing loss (Kikkawa and Miyasaka, 2016). Therefore, C57BL/6J mice are widely used as age-related hearing loss model. In a previous study, we have performed serial hearing measurements and analyzed hair cell survival in knockout and control mice from a C57BL/6J background at increasing ages (Cortada et al., 2023). For the current review, we have re-analyzed data from control mice to precisely investigate appearance of hearing loss and hair cell loss over age (Figure 2). Auditory brainstem response (ABR) and distortion-product otoacoustic emissions (DPOAE) hearing thresholds were already significantly increased at 8–12 weeks of age in higher frequencies (Figures 2A,B), as is known for mice from a C57BL/6J background (Bowl and Dawson, 2015; Kikkawa and Miyasaka, 2016). At 12 weeks of age, mice also showed outer hair cell loss at the cochlear base, whereas there was no inner hair cell loss at the investigated ages (Figures 2C,D). However, outer hair cells were only lost in frequency regions above 45 kHz, and therefore outside of our tested frequency range (Figure 2D). Therefore, the hearing loss observed (in the tested frequency regions) is not due to hair cell loss. Notably, 12 weeks old mice showed already elevated DPOAE hearing thresholds when compared with 2 week old mice at higher frequencies (Figure 2B). DPOAE are a measure of outer hair cell function. Given that outer hair cell loss is not the reason for the elevated DPOAE hearing thresholds (Figure 2D), these results suggest outer hair cell dysfunction. These results are in line with the aforementioned study by He and colleagues, which showed that a functional decline of hair cells precedes hair cell loss in age-related hearing loss (Liu et al., 2022). Therefore, age-related hearing loss precedes hair cell loss, which is not the primary reason for hearing loss.

Another relevant form of acquired hearing loss is noise-induced hearing loss. Depending on the intensity and duration of the noise trauma, noise-induced hearing loss leads to either temporary hearing threshold shifts or permanent hearing threshold shifts. In temporary threshold shifts, hearing thresholds fully recover within days to weeks after noise trauma. With longer duration and increasing intensity of noise trauma, hearing only recovers incompletely leading to permanent hearing threshold shifts, reflecting permanent cochlear damage. Very intense noise can directly cause mechanical trauma to hair cells, such as disrupting stereocilia (Ryan et al., 2016). Acute noise trauma leads to disrupted stereocilia and a moderate loss of synapses, but no hair cell loss (Gratias et al., 2021). Similarly, a study assessing the precise appearance of cytological defects in the cochlea upon a wide range of different noise exposure protocols showed an immediate damage to stereocilia upon noise damage, which was also the best predictor of degree and pattern of permanent hearing loss (Wang et al., 2002). Notably, hair cell loss appeared only later and permanent hearing loss was observed in cochlear regions without any significant hair cell loss (Wang et al., 2002). It has also been established that in addition to stereocilia especially the synaptic connections between inner hair cells and the auditory nerve are among the most vulnerable structures [reviewed in (Liberman and Kujawa, 2017)]. Synapse loss, also referred to as cochlear synaptopathy, is a major cause of hearing loss and occurs prior to hair cell loss (Liberman and Kujawa, 2017). More likely to occur even before the aforementioned cellular defects are molecular events in hair cells upon noise damage. Advancements in OMICS techniques allow for broad analysis of molecular events in hearing loss, such as noise-induced hearing loss. Savas and colleagues performed a proteomics study in noise exposed mice using different noise exposure protocols inducing permanent and temporary hearing loss (Jongkamonwiwat et al., 2020). The different noise exposure protocols used showed no substantial hair cell death. However, they observed a reduction in synapse numbers as well as disrupted stereocilia upon noise damage (Jongkamonwiwat et al., 2020). Using a proteomics approach they showed an acute accumulation of many proteins, such as cytoskeletal, heat shock, and proteasomal proteins, and reduction of collagens upon noise-exposure (Jongkamonwiwat et al., 2020). Protein ubiquitylation acutely increased in a noise intensity-dependent manner whereas the protein synthesis machinery was upregulated in the recovery period (2 weeks after noise), which was observed more in temporary than in permanent hearing loss (Jongkamonwiwat et al., 2020). Another study used a transcriptomic approach to investigate noise-induced cellular events in the cochlea (Milon et al., 2021). Although there was significant hearing loss in the tested frequency range until 32 kHz, there was no outer hair cell loss in the same cochlear region upon noise exposure (Milon et al., 2021). Many noise-induced transcriptional changes detected were cell type specific. Interestingly, mRNA metabolism related genes were downregulated in outer hair cells upon noise exposure (Milon et al., 2021). These studies clearly indicate that molecular and functional changes underlie noise-induced hearing loss, which precede the loss of hair cells.

Drug-induced hearing loss is another important form of acquired hearing loss. Different drugs can cause either temporary or permanent hearing loss via different mechanisms. For instance, salicylates can bind to the motor protein PRESTIN, impairing cochlear amplification and causing hearing loss (Sheppard et al., 2014). Other important drug classes causing hearing loss are platinum-based chemotherapeutic agents such as cisplatin and aminoglycoside antibiotics. Both cisplatin and aminoglycosides are taken up by hair cells leading to sustained accumulation in these sensory cells (Breglio et al., 2017; Jiang et al., 2017). Both drug categories are known as ototoxic eventually leading to hair cell loss involving mechanisms such as oxidative stress and apoptotic cell death pathways. Nevertheless, when performing electrophysiological analyses and analyzing cellular defects induced by cisplatin, Bao and colleagues found that cisplatin induced a significant delay of ABR wave 1 latency prior to hearing threshold elevations and hair cell loss (Chen et al., 2021). These changes where observed as early as after one treatment cycle with cisplatin, whereas with increasing cisplatin cycles hearing thresholds started to increase and hair cell loss was observed (Chen et al., 2021). Aminoglycoside antibiotic treatment can also lead to significant hearing threshold elevations in cochlear regions where no hair-cell loss is detectable (Nicol et al., 1992; Koo et al., 2015; Jiang et al., 2017). Further studies showed synaptic dysfunction and synapse loss prior to hair cell loss in aminoglycoside-induced hearing loss (Liu et al., 2013; Li et al., 2024). Therefore, even for classical drugs classified as ototoxic, an initial dysfunction of hair cells might be the underlying cause of hearing loss, secondarily leading to hair cell loss.

A detailed discussion of the molecular mechanisms leading to acquired hearing loss is not the scope of this review. Advances in molecular research on age-related, noise-induced, and drug-induced hearing loss are likely to reveal specific underlying mechanisms for each form of acquired hearing loss. However, it is likely that downstream mechanisms converge, since damage to hair cell elicits common cellular and molecular defects, such as stereocilia and synapse damage, as well as oxidative stress eventually converging on cell death mechanisms such as apoptotic signaling cascades [reviewed in (Wang and Puel, 2018)]. Future studies identifying early molecular events leading to acquired hearing loss will hopefully allow for specific intervention before irreversible damage and secondary loss of hair cells occur, allowing for better hearing rehabilitation.

### Human studies and hair cell loss

The inner ear is embedded within the temporal bone and contains a limited number of cells that are essential for hearing and balance. Consequently, human studies are constrained by limited accessibility and the impossibility of obtaining tissue samples during life. Human temporal bone studies are conducted post-mortem, and early functional or morphological pathologies in hair cells might be missed, whereas hair cell loss can be assessed. For these reasons, animal studies are useful to study the complex pathological features of hearing loss and early functional deficits. However, for translational relevance, it is also important to consider human studies. In this section, we review supporting evidence for secondary hair cell loss in human studies.

In pioneering work, Schuknecht described four predominant types of human age-related hearing loss, which is also named presbyacusis: Sensory presbyacusis from cochlear hair cell loss, neural presbyacusis from loss of cochlear neuronal structures, strial or metabolic presbyacusis from atrophy of the stria vascularis, and indeterminate presbyacusis with no obvious pathologic correlate (Schuknecht and Gacek, 1993; Merchant and Nadol, 2010). Studies focusing on cellular pathology in the inner ear alone might miss more subtle subcellular or functional defects. From animal studies and as reviewed above, it is known that major early morphological changes contributing to hearing loss are, stereocilia pathology and synapse degeneration. Stereocilia pathology in aging human temporal bones has been appreciated (Nomura and Kawabata, 1979; Bullen et al., 2020). Wu, Liberman et al. have performed seminal studies employing human temporal bone histology to investigate pathological changes in individuals with hearing loss, revising also previous studies performed by Schuknecht and colleagues (Wu et al., 2019; Wu et al., 2020a; Wu et al., 2020b; Wu et al., 2021; Wu and Liberman, 2022). While Schuknecht and colleagues proposed that most presbyacusis forms in humans are metabolic and/or neural (Merchant and Nadol, 2010), Wu, Liberman and colleagues showed that hearing loss is better predicted by hair cell loss (Wu et al., 2020a). However, this study also suggested hearing loss by uncaptured effects, which might be due subtler pathologies or functional defects (Wu et al., 2020a). Notably, in a subsequent study the same group also showed stereocilia pathology in the human cochlea, showing decreased stereocilia regularity and increased stereocilia loss with age (Wu and Liberman, 2022). Remarkably, in the only case where an audiogram was available, stereocilia pathology correlated better with hearing loss than hair cell death (Wu and Liberman, 2022).

In addition to stereocilia pathology, cochlear synaptopathy has also been shown in human presbycusis. Age-related loss of auditory nerve fibers was already noted in early observations (Felder and Schrott-Fischer, 1995). When applying the same synaptic staining protocols than used in animal studies, it was also possible to show that cochlear synaptopahty is a pathologic feature of human presbycusis (Viana et al., 2015). Given post-mortem autolysis of the synaptic region, a larger study focused on the analysis of auditory nerve fibers (Wu et al., 2019). Notably, the age-related loss of auditory nerve fibers exceeded the loss of hair cells by almost 3:1, and in most patients >50 years of age over 50% of auditory nerve connections had degenerated (Wu et al., 2019). In addition to age, a history of noise exposure exacerbates the loss of auditory nerve fibers in human temporal bones (Wu et al., 2021).

Another interesting example of secondary hair cell loss in humans comes from patients with *Otof* mutations. As reviewed earlier, *Otof* mutations cause an auditory synaptopathy. Hearing loss is due to defective synaptic transmission between inner hair cells and the spiral ganglion neurons constituting the auditory nerve. Otoacoustic emissions are a measure of outer hair cell function, and typically present in patients with *Otof* mutations. However, studies have shown that otoacoustic emissions deteriorate over time in patients with *Otof* mutations, indicating secondary outer hair cell degeneration upon hearing loss (Rodriguez-Ballesteros et al., 2003; Kitao et al., 2019). These studies are in line with similar observations from mouse studies (Stalmann et al., 2021).

To conclude, human temporal bone studies show hair cell death in both noise-induced and age-related hearing loss (McGill and Schuknecht, 1976; Kusunoki et al., 2004; Wu et al., 2020a). As demonstrated by the mouse studies reviewed above, secondary hair cell degeneration following the onset of hearing loss can occur relatively early in the disease course. This temporal sequence indicates that structural hair cell loss may follow an initial phase of functional impairment rather than represent the primary pathogenic event. Accordingly, hair cell loss observed in human temporal bones may likewise reflect a downstream consequence of earlier cellular dysfunction, particularly given that these tissues are analyzed post-mortem, often long after the initial onset of hearing loss. In addition, there is emerging evidence in human temporal bone studies of more subtle defects upon hearing loss, such as stereocilia pathology and synaptopathy. Lastly, functional studies in humans with genetic hearing loss are also indicative for secondary hair cell degeneration upon hearing loss.

### Hair cell loss as a direct cause of hearing loss

As we have reviewed above, hair cell loss represents often a downstream consequence of dysfunction of hair cells themselves or other functional deficits in the cochlea. Therefore, hair cell loss is often secondary to hearing loss. Nevertheless, there are also exceptions, where hair cell loss seems to be primary, i.e., where hair cell loss could be the underlying cause of auditory dysfunction. For instance, mutations that directly affect apoptosis-related pathways in hair cells are expected to cause primary hair cell loss responsible for hearing loss. The *DFNA5* gene was discovered to cause autosomal dominant hearing loss (Van Laer et al., 1998). The gene was later found to encode an apoptosis-inducing protein (Op de Beeck et al., 2011). Thus, hearing loss is expected to result from hair cell death after a gain-of-function mutation in the apoptosis inducing DFNA5 protein (Op de Beeck et al., 2011). Similarly, a genomic duplication of the tight junction protein gene *TJP2* was found to cause the autosomal dominant, adult-onset, progressive nonsyndromic hearing loss form DFNA51 (Walsh et al., 2010). Mechanistically, this genomic *TJP2* duplication was shown to reduce phosphorylation of GSK-3β at Ser9 and change the expression of apoptosis-related genes, which was proposed to cause hearing loss due to hair cell death in DFNA51 patients (Walsh et al., 2010). *TJP2* encodes the tight junction protein TJP2 (ZO-2). The effect of this mutation on the stability of intercellular junctions in the cochlea and whether a tight junction dysfunction triggers apoptosis remains unclear.

## Discussion and conclusion

**FIGURE 3:**
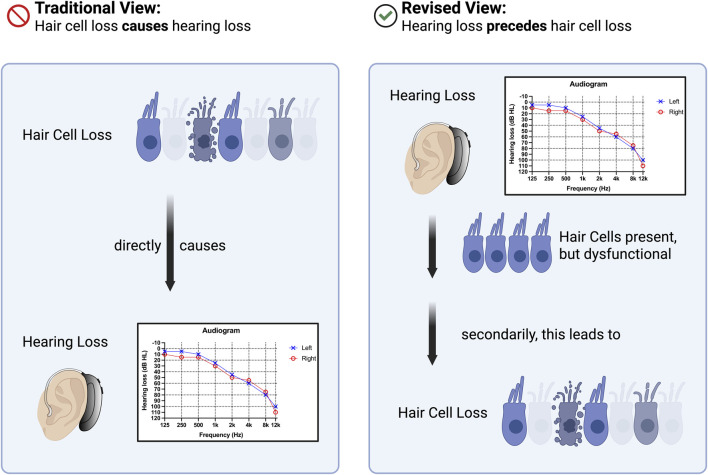
Hair cell loss–cause or consequence of hearing loss? Traditionally, hair cell loss has been considered a primary cause of hearing loss. However, a revised perspective suggests that hearing loss precedes hair cell loss, with the latter occurring as a secondary consequence.

Hair cell loss has historically been emphasized as a major mechanism underlying hearing loss and is still referred to in the current literature (McGovern and Cox, 2025). After decades of research, other mechanisms such as subtle morphological changes in hair cells or cochlear metabolic dysfunction have also been recognized as contributors to hearing loss. As we outlined in the current review, substantial evidence from genetic studies (Steel, 2011), from acquired forms of hearing loss, and also from human studies shows, that hair cell loss is often merely secondary to hearing loss and loss of hair cell function (Figure 3).

The mechanisms underlying secondary hair cell loss upon hearing loss remain incompletely understood. It might be that a common molecular hair cell dysfunction triggers hair cells to die [e.g., calcium homeostasis (Fettiplace and Nam, 2019)], or there might be specific underlying mechanisms for each form of hearing loss. The loss of hair cells occurs secondary to mutations that impair the function of hair cells. The mutations either affect the hair cells themselves or affect only non-sensory cells, which subsequently impair normal hair cell function. In both scenarios, functional impairment precedes structural degeneration, suggesting that the cochlea may eliminate sensory hair cells that are non-functional or persistently inactive. Notable exceptions exist in which hair cell loss seems to be the primary cause of hearing loss, such as mutations that directly affect apoptosis-related pathways in hair cells. Different mutations in the same gene that disrupt hair cell function can lead to different levels and timing of secondary hair cell death (Manji et al., 2012). The underlying reasons for this differential vulnerability and temporal progression of secondary hair cell death remain unknown. An effect of the genetic background on the severity of secondary hair cell loss has been shown (Cheatham et al., 2015).

Why are unfunctional hair cells eliminated in the cochlea after the occurrence of hearing loss? Hair cells are highly metabolic active with substantial energy consumption and also have a large total mitochondrial volume (McQuate and Raible, 2023). From an evolutionary perspective, it seems logical that the cochlea has quality control mechanisms in place to eliminate dysfunctional, energy consuming hair cells (Moreno and Rhiner, 2014). The cochlea is a highly complex microenvironment. For proper hearing function, not only a molecular homeostasis and mechanisms for ion buffering are required, but also highly specific mechanical properties must be maintained. Dysfunctional hair cells might impair both molecular processes, such as calcium buffering or the ion homeostasis, and disrupt mechanical properties unfavorable for proper hearing function. The secondary hair cell loss after hearing loss might therefore also be a (mis)adaptation of the cochlea in an effort to maintain hearing function. Similarly, hair cell loss might be a protective mechanism against tinnitus, which can develop following hearing loss due to maladaptive neural plasticity [reviewed in (Shore et al., 2016; Wu et al., 2016)].

The recognition that hair cell death most often occurs only secondarily to hearing loss has important therapeutic implications. Gene therapy to correct for defective hair cell genes needs to occur before hair cells are lost, which cannot be regenerated. This therapeutic window is a major limitation for the ongoing development of gene therapies. Similarly, for acquired hearing loss forms, understanding that hair cell dysfunction precedes the loss of hair cells allows for intervention and correction of hair cell dysfunction prior to loss of hair cells. The regeneration of lost hair cells is currently challenging. Therapies purely focusing on the intervention to block apoptotic pathways in hair cells might lead to hair cell preservation, but hair cells might still be unfunctional. Therefore, the focus on interventions to reactivate hair cell function prior to hair cell loss might be an interesting therapeutic strategy against hearing loss to explore. Such approaches could include strategies to preserve mechanoelectrical transduction efficiency, synaptic function, outer hair cell performance, mitochondrial health, metabolic function, calcium homeostasis or supporting cell function.

Lastly, in studies of genetic and acquired hearing loss, it is important to precisely analyze the underlying cause of hearing loss and hair cell loss at the earliest occurrence of hearing loss, in a frequency specific manner. Otherwise, secondary hair cell loss might be misinterpreted. It is important to note that experimental models such as mice exhibit a hearing range that extends well into the ultrasonic frequency spectrum, a region that is often insufficiently explored, given that most commercial speakers have an upper frequency limit of approximately 32 kHz. In addition, functional hearing assessment should extend beyond the measurement of auditory thresholds. In both clinical and experimental settings, hearing thresholds represent a fundamental metric and are a reliable predictor of hair cell loss (Ryan and Dallos, 1975; Stebbins et al., 1979; Wu et al., 2020a). However, threshold measurements may fail to detect earlier or more subtle forms of auditory dysfunction. Therefore, comprehensive auditory evaluation should extend beyond threshold measurements, such as analysis of the suprathreshold amplitude of ABR wave I as a measure of cochlear synaptopathy (Liberman and Kujawa, 2017), as well as assessment of shifts in place-frequency coding measured by DPOAE group delays (Kimberley et al., 1993; Zhang et al., 2022).

To conclude, hair cell loss is commonly a secondary event in both genetic and acquired forms of hearing loss. Thus, it is not a primary cause but rather a consequence of hearing loss.
